# Slamming dynamics of diving and its implications for diving-related injuries

**DOI:** 10.1126/sciadv.abo5888

**Published:** 2022-07-27

**Authors:** Anupam Pandey, Jisoo Yuk, Brian Chang, Frank E. Fish, Sunghwan Jung

**Affiliations:** ^1^ Biological & Environmental Engineering Department, Cornell University, Ithaca, NY 14853, USA.; ^2^ Cambridge Design Partnership, Raleigh, NC 27603, USA.; ^3^ Department of Biology, West Chester University, West Chester, PA 19383, USA.

## Abstract

In nature, many animals dive into water at high speeds, e.g., humans dive from cliffs, birds plunge, and aquatic animals porpoise and breach. Diving provides opportunities for animals to find prey and escape from predators and is a source of great excitement for humans. However, diving from high platforms can cause severe injuries to a diver. In this study, we demonstrate how similarity in the morphology of diving fronts unifies the slamming force across diving animals and humans. By measuring a time-averaged impulse that increases linearly with the impact height, we are able to estimate the unsteady hydrodynamic forces that an average human body experiences during the slamming phase of a feet-first, hand-first, or head-first dive. We evaluate whether the unsteady forces put the diver at risk of muscle or bone injuries for a particular diving height. Therefore, this study sheds light on a hydrodynamics-based protocol for safe high diving and an evolutionary driver for animal morphology.

## Teaser

This study investigates how humans and animals are injured due to diving.

## INTRODUCTION

The apparently mundane phenomenon of a solid object impacting the surface of water at high speeds is at the heart of many fascinating observations that range from the curious case of a stone skipping across the surface of a pond (*1*, *2*), the aesthetically pleasing, splash-less “rip” entry of Olympic divers (*3*, *4*), to the remarkable sight of hundreds of gannets plunging into the sea (*5*, *6*). The interest of the scientific community in the problem of water entry was initially fueled by its potential application in the design of warheads during the Second World War (*7*, *8*). These studies were based on von Karman’s investigation in 1929 regarding seaplane landing boats (*9*). At a much smaller length scale, analogous hydrodynamics explain how a lizard walks on water; the basilisk lizard creates a cavity with each step, pushes on the side of the cavity wall to move forward, and pulls out its foot before the cavity collapses (*10*). A similar physical insight can also be applied to sports performance research, such as the water entry of divers (*11*) and improving the design of oars in rowing (*12*).

As an object pierces the air-water interface and moves through the liquid bulk, it exhibits rich phenomenological events: splashing, cavitation, pinch-off, and rippling, which are spread across multiple length and time scales (*13*). These hydrodynamic events emerge from the competition of inertia, surface tension, gravity, and viscosity. While unsteady, inertial fluid forces are dominant during the slamming stage when the impacting body penetrates the water surface. Hydrostatic and viscous forces slow down the dynamics at later times. Measurement of the dynamic impact force shows a rapid increase in magnitude immediately after impact, leading to large values of impulse that can cause severe injuries. Plunge-diving birds such as gannets and boobies use the dense layer of feathers behind their head to transmit the impact force to their long, flexible neck that bends in response (*14*). Humans, however, without any anatomical feature to cushion their body, are prone to diving-related injuries. Recreational diving is one of the main causes of head and spinal cord injuries in the United States (*15*–*17*). Conventional wisdom held by professional divers is to jump feet first for heights above 10 m (*11*). Amateur divers and participants of recreational sports such as “death diving,” on the contrary, often hit the water surface with the head first to mimic death-defying stunts and excite the spectators. These observations raise the following questions: How much force does the impacting front experience? How does that force vary across different diving postures? When does the force reach the critical limit of causing injury?

**Fig. 1.**
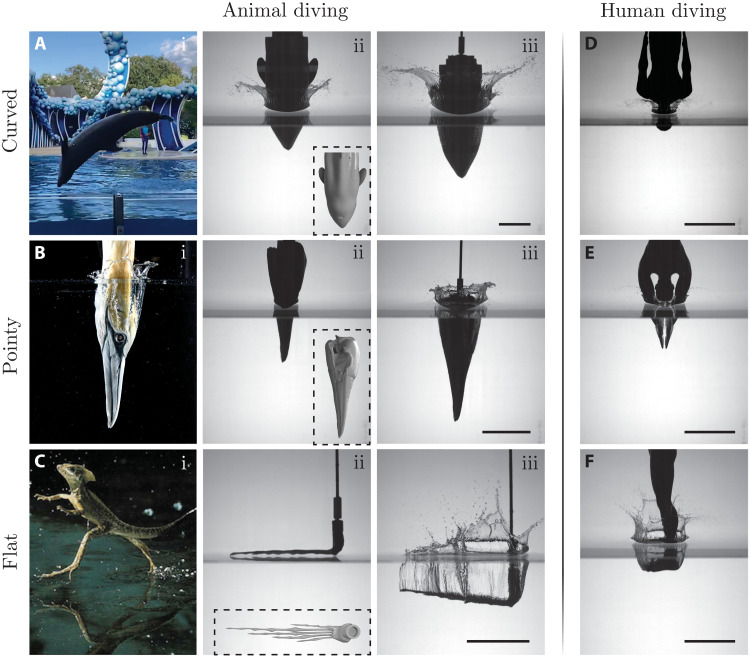
Impact dynamics in animal and human diving. We categorize the diving form of humans and diving or walking animal bodies into curved (first row), conical (second row), and flat (third row). (**A**) (i) Snapshot of a dolphin just before water entry while performing acrobatics in an aquarium. The curvature of their snout is the key parameter dictating the impact force on the head. (ii and iii) Laboratory experiment of an impacting porpoise head. (**B**) (i) Water entry of a plunge-diving gannet [this panel has been adopted from (*6*)]. A small beak angle ensures a slow increase in the impact force. (ii and iii) Experimental snapshot of a 3D printed gannet skull piercing through the water surface. (**C**) (i) Water-walking basilisk lizard [image credit: Stephen Dalton; this panel has been adopted from (*35*)]. As the flatfoot slaps against the water, a large cavity forms immediately. (ii and iii) A model of the lizard foot. (**D** to **F**) Impact of 3D printed human models in head-first, hand-first, and feet-first forms, respectively. Insets are the AutoCAD rendering of the models. Scale bars, 10 cm. All of the images correspond to a drop height of 1 m.

Here, we study the slamming dynamics of animal and human dives and show how the forces of slamming lead to diving-related injuries. The impulsive nature of these forces makes them harder to be absorbed by muscles and tissues, and consequently, a diver becomes prone to suffer from injuries. Since an injury at the slamming phase might impair the ability of the diver to perform necessary maneuvers to safely reach the surface, understanding the hydrodynamic forces of this phase is critical. We examine how the overall shape of diving fronts unifies the slamming forces in plunge-diving animals and humans diving in different postures. In this regard, we use three-dimensional (3D) printed models of a harbor porpoise (*Phocoena phocoena*) head, Northern gannet (*Morus bassanus*) beak, and basilisk lizard (*Basiliscus basiliscus*) foot as representative of curved, conical, and flat projectiles, respectively. For human dives, the analogous forms are head-first, hand-first, and feet-first configurations (cf. Fig. 1) where the diving front is curved, pointy, and flat, respectively. We find that the classical added mass forces of unsteady hydrodynamics are able to capture the measured forces on animal and human models, revealing the role of the morphology of a diving front on the early time dynamics. We incorporate the temporal evolution of the measured force into a time-averaged impulse that quantifies an effective slamming force for a given front shape and size. This effective slamming force is found to scale linearly with the height of the dive. Thus, whenever the diving height is such that the slamming force exceeds the critical compressive strength of muscles, ligaments, or bones, injuries occur. Through our hydrodynamic analysis, we conclude that the human upper torso, including the cervical spine and collarbone, is vulnerable to injuries at heights above 8 and 12 m when diving head and hands first, respectively. However, when diving feet first, the critical height of injury is about 15 m.

## RESULTS

### Experimental observations

3D printed animal and human models, connected to a force sensor, are dropped into a water tank under gravity. A slider, on which the force sensor is mounted, ensures a vertical, free fall impact of different models with impact velocity, $V≃\sqrt{2\mathrm{gh}}$ (see Materials and Methods for details). We record the dynamic force at a rate of 2000 Hz. A high-speed camera synchronized with the force sensor helps us measure the impact speed as a structure moves through the water. Typical snapshots from experiments are shown in Fig. 1, which are arranged into three rows: The first row shows porpoise and human head-first impacts, which belong to the curved category (cf. Fig. 1, A, i to iii, and D). The second row shows gannet and human hand-first impacts, which belong to the conical/pointy category (cf. Fig. 1, B, i to iii, and E). The third row shows lizard and human feet-first impacts, which fall into the flat category (cf. Fig. 1, C, i to iii, and F). Here, we would like to point out that trained divers opt for thumb-in-palm or flat-hand grab postures while diving hand first to reduce splash and better protect the wrists and arms from injury (*18*, *19*). Cliff divers, during feet-first entry, adopt either flexed, horizontal feet as shown in Fig. 1F or angled (downward) feet with flexed toes. Our choice of human configurations was motivated by the similarity in overall shape with diving animals.

**Fig. 2.**
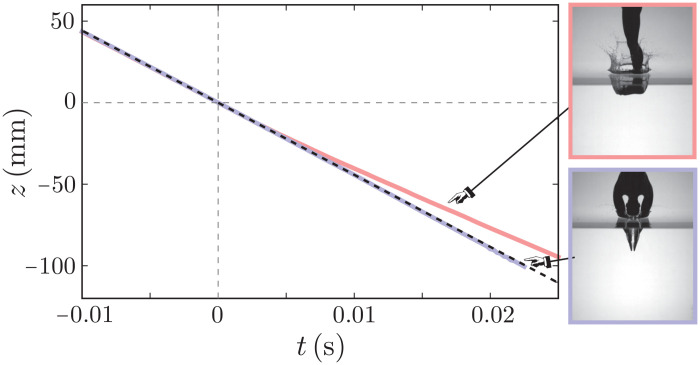
Kinematics of slamming. Position versus time plot for hand-first (blue line) and feet-first (red line) dives. Both models were dropped from a height of 1 m, reaching an impact velocity of 4.41 m/s. The theoretical free fall velocity ($\sqrt{2\mathrm{gh}}=4.43$ m/s) is plotted as the black dashed line. Both the position and time are measured from the water surface.

In an ideal dive, where the diver enters the water smoothly without any harsh consequence, the kinetic energy of impact is slowly dissipated by the flow. At early times, however, the force of impact on the body comes from the rate of change of added mass of water. Consequently, velocity remains constant at the impact value. This behavior is confirmed by the position versus time data of the hand-first model shown in Fig. 2; a free fall trajectory (black dashed line) perfectly captures the measured positions under water (blue line). Both curved and pointy structures exhibit this constant velocity phase at early times. Feet-first models, on the contrary, create a large cavity upon impact and trigger splashing. Thus, the structure slows down immediately after slamming, as shown by the red line in Fig. 2. This qualitative difference between different diving postures is captured in the dynamic force measurements, which we discuss in detail in the following sections.

### Unsteady slamming force

The impact dynamics of projectiles involve inertial, viscous, surface tension, and gravitational forces. Balancing the strength of the inertial force with the rest, we obtain three dimensionless numbers: Re, We, and Fr. Our experiments are performed in Re > 10^4^, We > 10^3^, and Fr ≃ 1 − 10 (see table S1 for details). Thus, the inertial and gravitational forces are of comparable strength. In the slamming regime, the velocity of a projectile remains constant, and thus, the slamming force (*F*) is dominated by an unsteady hydrodynamic force, *F* = *V*^2^d*m*_a_/d*z*, where *m*_a_ is the added mass carried by the body and *V* is the impact velocity (*20*, *21*). Using von Karman’s simplified model (*9*), the added mass term, *m*_a_, can be written as the hemispherical liquid volume of the radius equal to the cross-sectional radius of the body at the water surface. Hence, the impact force simplifies as

$$F=\alpha \rho V^{2}r^{2}\frac{dr}{dz}$$ (1)

Here, ρ is the density of water. This equation demonstrates how the impact force depends on the body shape [*r*(*z*)], with *z* being the penetration depth. The constant of proportionality (α) depends on the nature of the flow field around the body (*22*), and thus, it is different for different shapes. In Eq. 1, we use the constant velocity assumption as *z* = *Vt*. In the rest of the paper, we will show how the force-time relation predicted from Eq. 1 captures the slamming dynamics of animal and human dives.

**Fig. 3.**
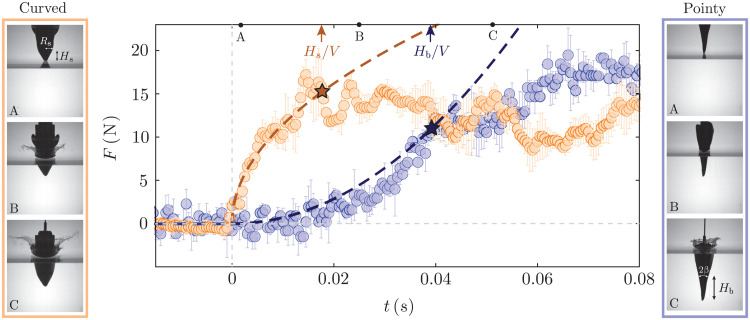
Evolution of the slamming force for harbor porpoise and gannet heads. Data points represent experimental measurements, whereas dashed lines are analytical predictions. The porpoise head (orange data) is dropped from a height of 60 cm, reaching an impact velocity of 3.42 m/s, while the gannet head data (blue points) correspond to a free fall height of 80 cm, reaching an impact speed of 3.95 m/s. The darker orange dashed line represents Eq. 2, whereas the darker blue dashed line represents Eq. 3. The star markers represent the end of the slamming phase marked by *H*_s_/*V* and *H*_b_/*V*, respectively. Force values shown in the figure have been averaged over five trials, and the error bars measure the SD in the measurement. The time stamps corresponding to the two sets of images are marked on the figure.

Force measurements for a curved (porpoise) and a conical (gannet) model are shown in Fig. 3. The time evolution of the impact force is very distinct for the two cases; the gannet head exhibits a smooth increase in force upon impact, while for porpoise head, a discontinuous transition leads to an immediate increase in force. The force increases in both cases until flow separation is triggered by irregularity in shape. Once the flow separates from the body, force-time behavior changes abruptly. We denote the phase between the touchdown and flow separation as the “slamming phase.” Stars on the data points of Fig. 3 mark the end of the slamming phase.

#### Diving porpoise

As mentioned before, the overall geometry of porpoise and gannet heads dictates the force response observed in Fig. 3. Leaving aside the geometric details of the two structures in section SI, here, we present the key features of the analytical prediction. We approximate the blunt snout of a porpoise as a paraboloid with its radius as *r* = (*z*/κ_m_)^1/2^, where the mean curvature of the snout at its tip, κ_m_, is estimated as $\kappa_{m}=2H_{s}/R_{s}^{2}$ with *H*_s_ and *R*_s_ being the height and base radius of the snout, respectively. Thus, Eq. 1 becomes

$$F=\alpha \rho V^{5/2}\kappa_{m}^{-3/2}t^{1/2}$$ (2)

where the prefactor, α, has been found to be $4\sqrt{2}$ (*23*). Equation 2 is plotted as the red dashed line in Fig. 3, which predicts the experimental data until the snout is fully submerged in water, given by the slamming time *t*_s_ = *H*_s_/*V*. At this instant, the slamming force reaches the maximum value of $F_{m}=4\sqrt{2}\rho V^{2}H_{s}^{1/2}\kappa_{m}^{-3/2}=2\rho V^{2}R_{s}^{3}/H_{s}$. Beyond *t*_s_, an air cavity forms, and the flow separates from the body.

#### Diving gannet

The impact force on the pointy beak of a gannet is reasonably well captured by a conical shape of a half-angle β (measured from the vertical axis) (*6*). Replacing *r* = *z* tan β in Eq. 1, we get an analytical expression of the impact force as

$$F=\alpha \rho V^{4}{\text{tan}}^{3}\beta t^{2}$$ (3)

where the numerical factor α = π (*24*). Thus, the smaller the cone angle, the slower the rate of increase in force. This quadratic relation between *F* and *t*, as shown by the blue dashed line in Fig. 3, explains the continuous transition of force as the body hits the water. The angle of the conical geometry is defined by tan β = *R*_b_/*H*_b_, with *H*_b_ and *R*_b_ being the height and base radius of the beak, respectively. Thus, the impact force at the slamming time *t*_s_ = *H*_b_/*V* reaches $F_{m}=\pi \rho V^{2}R_{b}^{3}/H_{b}$.

#### Diving hand first

**Fig. 4.**
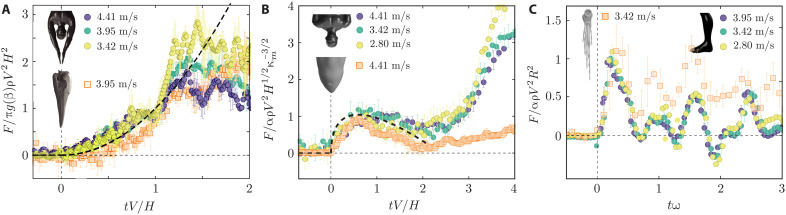
Dimensionless force versus time for the three different groups of models. (**A**) Gannet head and human hand-first impact follows the force-time relation of Eq. 3, which is captured by the dimensionless relation $\bar{F}=t^{-2}$, plotted as the black dashed line. The prefactor in the force scale is defined as *g*(β) = tan^3^β for the gannet head and *g*(β) = tan^3/2^β_1_tan^3/2^β_2_ for hand first. (**B**) Porpoise head and human head-first impact data are captured by Eq. 4, as represented by the dimensionless relation of $\bar{F}=(1.19\;\pi /8)(4\sqrt{2\bar{t}}-1.19\pi \bar{t})$ (black dashed line). The numeric factor α is $4\sqrt{2}$ for the porpoise head and 8/(1.19π) for the human head. (**C**) Human and lizard foot impact shows high-frequency oscillations in the force data. Time axis is scaled by the resonance frequency (ω) of the bubble formed underneath the foot. The force values are scaled by the inertial force scale *αρV*^2^*R*^2^, where the numerical prefactor α = 11.

Here, we show how the slamming dynamics of curved and conical bodies allow us to estimate impact forces in human diving with different postures. Amateur divers often dive with both arms extended above the head, bringing the palms together in a posture that we refer to as hand first. Diving in this hand-first orientation leads to a smooth water entry and prevents direct impact on the head. Dropping the hand-first model from different heights, we notice that the force-time behavior bears a qualitative similarity with the slamming forces of the gannet beak (fig. S3A). For a comprehensive comparison between the two, here, we present the dimensionless data that incorporate different velocities and geometries. Introducing dimensionless time $\bar{t}=\mathrm{tV}/H$ and force $\bar{F}=F/(\pi {\text{tan}}^{3}\beta \rho V^{2}H^{2})$, we get a universal force-time relation as in Eq. 3 of the form $\bar{F}={\bar{t}}^{2}$. Since the hand-first posture is not axisymmetric, we use a mean radius *r* = *z*( tan β_1_ tan β_2_)^1/2^ to express the slamming force that is equivalent to Eq. 3. Here, β_1_ and β_2_ are the two wedge angles in the coronal and sagittal planes, respectively (fig. S1E). Thus, the dimensionless force for the hand-first dive takes a slightly different form, $\bar{F}=F/(\pi {\text{tan}}^{3/2}\beta_{1}{\text{tan}}^{3/2}\beta_{2}\rho V^{2}H^{2})$. Figure 4A confirms that the above rescaling collapses the hand-first and gannet data onto one curve of $\bar{F}={\bar{t}}^{2}$, marked by the black dashed line during the slamming phase ($\bar{t}<1$).

#### Diving head first

Head-first dives, where the skull is slammed against water, mostly occur during accidental falls and could lead to fatal brain injuries. The slamming force exhibits a nonmonotonic behavior, reaching a peak immediately after the impact. At very low penetration depths, the shape of the sphere is well approximated by a paraboloid, and one expects that the slamming force is captured by Eq. 2. This approximation breaks down even at moderate penetration depths of *z* ≥ 0.1*R*, where the slope of a spherical surface (d*r*/d*z*) rapidly falls to zero. The peak force is much smaller than what is predicted by Eq. 2 (see figs. S3B and S4). This discrepancy is due to the fact that the flat plate approximations of both von Karman and Wagner theories overestimate the pressure in the limit of small d*r*/d*z* (*7*, *8*, *25*). Once the “exact” spherical boundary is considered in the potential flow theory, to calculate the flow field and pressure, the slamming force becomes

$$F=4\sqrt{2}{\rho \kappa }_{m}^{-3/2}V^{5/2}t^{1/2}-1.19{\pi \rho \kappa }_{m}^{-1}V^{3}t$$ (4)

where κ_m_ = 1/*R* (*23*). One immediately notices that the first term in Eq. 4 is exactly the same expression of Eq. 2. The second term of Eq. 4 ensures a peak force of $F_{m}=8/(1.19\pi )\;\rho V^{2}\kappa_{m}^{-2}$ at *t* = 8/(1.19π)^2^ 1/(κ_m_*V*) ≃ 0.572/(κ_m_*V*). Rescaling the time by 1/(κ_m_*V*) and force by *F*_m_, we collapse the force-time curves for different velocities to a single master curve, given by $\bar{F}=(1.19\pi /8)(4\sqrt{2}{\bar{t}}^{1/2}-1.19\pi \bar{t})$, as shown by the black dashed line in Fig. 4B. The porpoise force data have been rescaled by its maximum force, $F_{m}=2\rho V^{2}R_{s}^{3}/H_{s}$, as discussed previously. Both force and time scales are shown for a generic curved body with two independent length scales of *H* and κ_m_. However, these expressions simplify for the head-first data with one length scale of radius $R(=\kappa_{m}^{-1}=H$).

#### Diving feet first

While the added mass increases continuously with submergence depth following the shape of conical and curved bodies, the impact of flat surfaces causes an instantaneous increase in the contact area between the solid and water, leading to a sharp spike in the force measurement. Thus, the slamming forces in this case cannot be understood by the analysis presented above. Experiments with 3D printed human and lizard foot (fig. S5) confirm the instantaneous peak force that scales with the impact velocity. To capture the peak slamming force, we use the generic inertial force scale of αρ*V*^2^*R*^2^ to rescale the force data. Here, *R* is the radius of an equivalent circular flat plate (see section SI for details of calculating *R*), and α is one-half of the instantaneous drag coefficient, which is chosen to be 22 in accordance with recent measurements on flat panels (*26*). In addition, the force data exhibit high-frequency oscillations, the period of which remains unchanged for all the impact velocities. Naturally, these oscillations have a different origin from slamming itself. Flat (and concave) surfaces trap air bubbles in the process of piercing the water surface, and the oscillations of these bubbles have been found to give rise to the fluctuations in the force measurements (*27*). Assuming that the concave arch supporting human feet traps a bubble of radius *w*_2_ (width of the feet model as shown in fig. S1F), we estimate the resonance frequency as $\omega =w_{2}^{-1}\sqrt{3\gamma P/\rho }≃109$ rad/s, where *P* is atmospheric pressure, γ( = 1.4) is the gas constant, and ρ is the water density (*28*). Figure 4C shows that a rescaling of time with ω perfectly captures the oscillation in force.

### Time-averaged impulse

The rapid increase in the magnitude of force upon impact could be fatal for human body as muscles and soft tissues are unable to absorb the short-interval, impulsive forces. The temporal evolution and magnitude of these slamming forces depend on the shape and size of an impacting body. We characterize the dynamics of slamming by the impulse it creates and estimate an effective force of slamming that is given by the time-averaged impulse

$$\langle F_{S}\rangle =\frac{\int_{0}^{t_{s}}Fdt}{t_{s}}$$ (5)

**Fig. 5.**
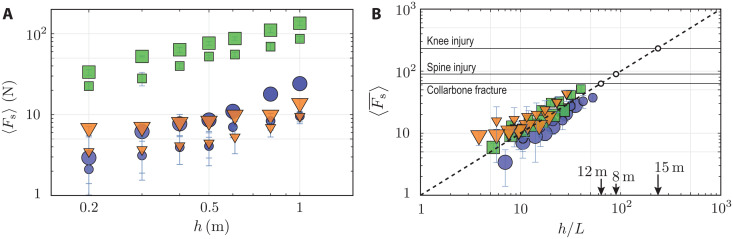
Variation of the time-averaged impulse with diving height. (**A**) Time-averaged impulse for hand-first (orange triangles), head-first (blue circles), and feet-first (green squares) impacts for different diving heights. Marker sizes represent data for two separate sizes of each model. (**B**) Rescaling the effective slamming force and impact height leads to the collapse of the data onto the master curve given by $\langle {\bar{F}}_{s}\rangle =h/L$, which is represented by the solid dashed line. Critical force values from Table 1 are plotted as the horizontal lines. The open circles represent critical heights for head-first (8 m), hand-first (12 m), and feet-first (15 m) dives causing injury. These dimensional heights correspond to an average body size (*L*) listed in Table 1.

For both hand-first and head-first dives, we integrate the experimental force up until *t*_s_ = *H*/*V* to determine ⟨*F_s_*⟩. However, for feet-first data, the slamming time (*t*_s_) is ill-defined. Thus, we estimate 〈*F*_s_〉 in this case by integrating the slamming force until it reaches the first peak in magnitude (see Fig. 4C). The resulting forces for the three different human diving postures are plotted against the diving height in Fig. 5A. Here, two different marker sizes represent data for two different sizes of human models. To extrapolate the slamming force from our experimental range to what an average human body might experience while diving, we rescale ⟨*F*_s_⟩ by α*ρ_f_gL*^3^ and the height *h* by *L*. This particular form of the characteristic force results from the integration of analytical prediction of *F*(*t*) and noting that *t*_s_ = *H*/*V* and $V=\sqrt{2\mathrm{gh}}$ (see section SIV for details). The shape factor α for the three diving postures are listed in Table 1, whereas the length scale *L* is chosen to be either the head radius (1/κ_m_), mean shoulder width (*w*_m_), or equivalent feet radius (*R*). Once rescaled, the data of Fig. 5A are collapsed onto one master curve given as

$$\langle {\bar{F}}_{S}\rangle =\frac{\langle F_{S}\rangle }{\alpha \rho {\mathrm{gL}}^{3}}=\frac{h}{L}$$ (6)

as shown in Fig. 5B. According to Eq. 6, the diving height (*h*) gives direct access to the effective force of slamming on the human body. Comparing this slamming force to the critical impact force responsible for injury, one can estimate critical diving heights at which injuries might occur.

**Table 1.** Injury associated to diving. Critical forces are nondimensionalized using the shape factor (α) and average adult body size (*L*). Values of α and *L* are discussed in section SIV. The lower limits of critical force values listed in the last column are plotted in Fig. 5B.

**Diving posture**	**Injury**	**Critical force (*F*_c_)**	**Typical size (*L*)**	**Shape factor (α)**	** ${\bar{F}}_{c}=\frac{F_{c}}{\alpha \rho gL^{3}}$ **
Head first	Cervical spine and neck (*36*) Skull fracture	2.4–5.9 kN (*37*, *38*) 4.2–6.2 kN (*39*, *40*)	Head radius (9.02 cm) (*29*)	$\left(\frac{16\sqrt{2}}{3}-1.19\pi \right)$	88–217 154–228
Hand first	Clavicular compression	2.06–3.43 kN (*41*) (for a 70-kg person)	Shoulder half-width (18.14 cm) (*42*)	0.54	65–109
Feet first	Knee Tibia fracture	8.24 kN (*43*) 7.9–16 kN (*45*)	Equivalent radius [7.04 cm based on (*44*)]	11	218 209–482

## DISCUSSION

In summary, we have rationalized the slamming force for human and animal dives by considering the unsteady hydrodynamic forces that depend on the diving-front shape of an impacting body. For diving animals, either a curved snout, a conical beak, or a flatfoot leads to a distinct force-time response as a consequence of the different added masses of the body. The same analysis captures the slamming force of hand-first, head-first, and feet-first dives of humans. By introducing a time-averaged impulse that is representative of the slamming dynamics, we are able to unify the measured forces across different shapes and sizes.

Making use of the dimensionless time-averaged impulse versus the diving height plot of Fig. 5B, we elucidate the question of what is a safe height for human diving in a particular body position. Since the hydrodynamic forces primarily exert compressive impact forces on the body, we use the critical compressive force available in the literature corresponding to human muscle and bone injuries. Table 1 presents a list of possible injuries, critical forces causing those injuries, and dimensionless values of the critical forces that are based on our analysis of slamming forces for the three different postures. Although different body parts are susceptible to injury during diving, we make the assumption that the head, neck, and cervical spine are the most vulnerable during head-first impacts, while bones and muscles in the limbs are prone to damage during hand-first and feet-first dives. As shown in Fig. 5B, these critical forces help us identify the maximum height above which diving leads to injury. For example, the lower limit of cervical spine injury corresponds to ${\bar{F}}_{c}=88$, which intersects the $\left\langle {\bar{F}}_{s}\right\rangle$ line at *h*/*L* = 88. Using the average human head radius (*L*) of 9.02 cm (*29*), we conclude that a height (*h*) ≃ 8 m is the critical height for causing spinal cord and neck injury with the head-first posture. Similarly, we find that hand-first dives from heights above 12 m may lead to collarbone injury, and feet-first dives from heights above 15 m are prone to cause injuries to the knee. Since humans can actively modulate their muscle recruitment and ligament tension, the above critical height predictions that are based on passive cadaver properties only provide conservative estimates. With increased strength and proper training, competitive cliff divers can safely dive from heights of 18 to 26 m. Estimates given here provide a safety guideline for amateur divers and diving enthusiasts.

While humans are prone to injury in diving from moderately high platforms, plunge-diving animals prevent injuries from repeated high dives through morphological adaptations and behavioral modifications. Northern gannets and brown boobies, for example, forage on fish by diving into water at speeds of 24 m/s (*30*). Both these species have comparatively shallow beak angle (β) relative to surface-diving birds (*31*), leading to much smaller slamming forces as $F∼{\text{tan}}^{3}\beta$. Furthermore, gannets dive with their necks held straight (*32*), placing the vertebrae in a columnar arrangement that reduces stresses on the ligaments and muscles of the neck (*6*). Dolphins (Delphinidae), which frequently porpoise, have developed an interesting morphological feature. The cervical vertebrae in these cetaceans are shortened and fused (*33*). This fused vertebra increases the stiffness of the overall structure supporting the bulbous head, making it resilient against the impact forces during porpoising (*34*).

## MATERIALS AND METHODS

### 3D modeling and 3D printing of objects

3D models are constructed in a stereolithography (STL) format based on computed tomography scan images using 3D Slicer software. Each STL file is modified to include a connector to a load cell through Fusion 360 software (Autodesk). For the human models, STL files are downloaded from clara.io (https://clara.io/view/d49ee603-8e6c-4720-bd20-9e3d7b13978a) and are modified to various diving postures using Blender software. For 3D printing, all models are sliced using Cura software (Ultimaker Ltd.) with 40 to 50% infill and 0.2-mm layer height. Sliced files are printed in Ultimaker S5 (Ultimaker Ltd.) with with Ultimaker thermoplastic polyester (2.85 mm NFC PLA) filaments.

### Experimental setup for diving experiments

Experiments are performed in a tank of 85 cm by 85 cm by 1 m. A slider is installed on a frame and is placed at the center of the tank. The slider is held in place with a latch connected to the frame. An LC101-25 (Omega Co.) load cell, capable of data collection at 2000 Hz, is mounted on the slider and is connected to the 3D printed models through a stainless steel rod 50 cm in length. Once the latch on the slider is released, the fixture combining the 3D model and the load cell free-falls to the water surface. The impact heights are varied between 20 and 100 cm to vary the impact speed. Signal from the load cell is amplified using a signal conditioner (2310B, Vishay) and collected through the Data Acquisition Card (USB-6001, NI). The voltage signals are converted to the force with a calibration curve. In front of the tank, we place a high-speed camera (Fastcam SAZ, Photron) with a 105-mm lens (Nikon) to capture the impact moment of the drops. The water tank is back-lit using light-emitting diode strips. All images are taken at 4000 frames per second. The high-speed camera and the load cell are synchronized with a trigger.

## Supplementary Materials

### This PDF file includes:

Sections SI to SIV Figs. S1 to S5 Table S1 References

## Notes

View/request a protocol for this paper from *Bio-protocol*.

## References

[1] C. Clanet, F. Hersen, L. Bocquet, Secrets of successful stone-skipping. Nature 427, 29 (2004). 14702075 10.1038/427029a

[2] T. Truscott, J. Belden, R. Hurd, Water-skipping stones and spheres. Phys. Today 67, 70–71 (2014).

[3] J. G. Brown, L. D. Abraham, J. J. Bertin, Descriptive analysis of the rip entry in competitive diving. Res. Q. Exerc. Sport 55, 93–102 (1984).

[4] H. Driscoll, S. Gaviria, S. Goodwill, Analysing splash in competitive diving. Procedia Eng. 72, 26–31 (2014).

[5] S. Garthe, S. Benvenuti, W. A. Montevecchi, Pursuit plunging by northern gannets (*Sula bassana*)" feeding on capelin (*Mallotus villosus*)". Proc. R. Soc. B Biol. Sci. 2670, 1717–1722 (2000). 10.1098/rspb.2000.1200 PMC1690745 12233767

[6] B. Chang, M. Croson, L. Straker, S. Gart, C. Dove, J. Gerwin, S. Jung, How seabirds plunge-dive without injuries. Proc. Natl. Acad. Sci. 113, 12006–12011 (2016). 27702905 10.1073/pnas.1608628113 PMC5087068

[7] A. May, J. C. Woodhull, Drag coefficients of steel spheres entering water vertically. J. Appl. Phys. 19, 1109–1121 (1948).

[8] A. May, J. C. Woodhull, The virtual mass of a sphere entering water vertically. J. Appl. Phys. 21, 1285–1289 (1950).

[9] T. Von Karman, *The Impact on Seaplane Floats During Landing* (National Advisory Committee on Aeronautics, 1929).

[10] J. W. Glasheen, T. A. McMahon, A hydrodynamic model of locomotion in the basilisk lizard. Nature 380, 340–342 (1996).

[11] T. Guillet, M. Mouchet, J. Belayachi, S. Fay, D. Colturi, P. Lundstam, P. Hosoi, C. Clanet, C. Cohen, The hydrodynamics of high diving. Proceedings 49, 73 (2020).

[12] R. Labbé, J.-P. Boucher, C. Clanet, M. Benzaquen, Physics of rowing oars. New J. Phys. 21, 093050 (2019).

[13] T. T. Truscott, B. P. Epps, J. Belden, Water entry of projectiles. Annu. Rev. Fluid Mech. 46, 355–378 (2014).

[14] K. Bhar, B. Chang, E. Virot, L. Straker, H. Kang, R. Paris, C. Clanet, S. Jung, How localized force spreads on elastic contour feathers. J. R. Soc. Interf. 16, 20190267 (2019). 10.1098/rsif.2019.0267 PMC6893494 31744417

[15] N. S. Jones, Competitive diving principles and injuries. Curr. Sports Med. Rep. 16, 351–356 (2017). 28902759 10.1249/JSR.0000000000000401

[16] S. M. Harrison, R. C. Z. Cohen, P. W. Cleary, S. Barris, G. Rose, Forces on the body during elite competitive platform diving, in *Ninth International Conference on CFD in the Minerals and Process Industries* (CSIRO, 2012), Melbourne, Australia, 10 to 12 December 2012.

[17] D. Wharton, Broken wrists, twisted necks and concussions: The brutal nature of olympic diving, *Los Angeles Times*, 29 July 2021.

[18] S. C. Haase, Management of upper extremity injury in divers. Hand Clin. 33, 73–80 (2017). 27886841 10.1016/j.hcl.2016.08.017

[19] D. T. le Viet, L. A. Lantieri, S. M. Loy, Wrist and hand injuries in platform diving. J. Hand Surg. Am. 18, 876–880 (1993). 8228062 10.1016/0363-5023(93)90058-B

[20] S. Abrate, Hull slamming. Appl. Mech. Rev. 64, 060803 (2011).

[21] S. Jung, Swimming, flying, and diving behaviors from a unified 2d potential model. Sci. Rep. 11, 15984 (2021). 34362958 10.1038/s41598-021-94829-7 PMC8346475

[22] H. Wagner, Phenomena associated with impacts and sliding on liquid surfaces. J. Appl. Math. Mech. 12, 193–215 (1932).

[23] T. Miloh, On the initial-stage slamming of a rigid sphere in a vertical water entry. Appl. Ocean Res. 13, 43–48 (1991).

[24] A. A. Korobkin, V. V. Pukhnachov, Initial stage of water impact. Annu. Rev. Fluid Mech. 20, 159–185 (1988).

[25] M. Moghisi, P. T. Squire, An experimental investigation of the initial force of impact on a sphere striking a liquid surface. J. Fluid Mech. 108, 133–146 (1981).

[26] F. J. Huera-Huarte, D. Jeon, M. Gharib, Experimental investigation of water slamming loads on panels. Ocean Eng. 38, 1347–1355 (2011).

[27] V. Mathai, R. N. Govardhan, V. H. Arakeri, On the impact of a concave nosed axisymmetric body on a free surface. Appl. Phys. Lett. 1060, 064101 (2015).

[28] M. Minnaert, On musical air-bubbles and the sounds of running water. Lond. Edinb. Dublin Philos. Mag. J. Sci. 16, 235–248 (1933).

[29] A. K. D. Nguyen, A. A. Simard-Meilleur, C. Berthiaume, R. Godbout, L. Mottron, Head circumference in Canadian male adults: Development of a normalized chart. Int. J. Morphol. 30, 1474–1480 (2012).

[30] Y. Ropert-Coudert, D. Grémillet, P. Ryan, A. Kato, Y. Naito, Y. L. Maho, Between air and water: The plunge dive of the Cape gannet *Morus capensis*. Ibis 146, 281–290 (2004).

[31] S. I. Sharker, S. Holekamp, M. M. Mansoor, F. E. Fish, T. T. Truscott, Water entry impact dynamics of diving birds. Bioinspir. Biomim. 140, 056013 (2019). 10.1088/1748-3190/ab38cc 31387087

[32] D. N. Lee, P. E. Reddish, Plummeting gannets: A paradigm of ecological optics. Nature 293, 293–294 (1981).

[33] E. A. Buchholtz, S. A. Schur, Vertebral osteology in delphinidae (cetacea). Zool. J. Linn. Soc. 140, 383–401 (2004).

[34] F. E. Fish, C. A. Hui, Dolphin swimming—A review. Mamm. Rev. 21, 181–195 (1991).

[35] A. E. Minetti, Y. P. Ivanenko, G. Cappellini, N. Dominici, F. Lacquaniti, Humans running in place on water at simulated reduced gravity. PLOS ONE 7, e37300 (2012). 22815681 10.1371/journal.pone.0037300 PMC3399875

[36] D. S. Korres, I. S. Benetos, G. S. Themistocleous, A. F. Mavrogenis, L. Nikolakakos, P. T. Liantis, Diving injuries of the cervical spine in amateur divers. Spine J. 6, 44–49 (2006). 16413447 10.1016/j.spinee.2005.06.013

[37] T. Whyte, A. D. Melnyk, C. Van Toen, S. Yamamoto, J. Street, T. R. Oxland, P. A. Cripton, A neck compression injury criterion incorporating lateral eccentricity. Sci. Rep. 10, 7114 (2020). 32346007 10.1038/s41598-020-63974-w PMC7189232

[38] F. Li, N.-s. Liu, H.-g. Li, B. Zhang, S.-w. Tian, M.-g. Tan, B. Sandoz, A review of neck injury and protection in vehicle accidents. Transp. Saf. Environ. 1, 89–105 (2019).

[39] S. Advani, W. Powell, J. Huston, S. Ojala, Human head impact response–experimental data and analytical simulations, in *Proceedings of the International Conference on the Biomechanics of Impact* (IRCOBI, 1975), pp. 153–162.

[40] D. C. Schneider, A. M. Nauhm, Impact studies of facial bones and skull, in *Proceedings: Stapp Car Crash Conference* (Society of Automotive Engineers SAE, 1972), vol. 16.

[41] D. Stanley, E. A. Trowbridge, S. H. Norris, The mechanism of clavicular fracture. A clinical and biomechanical analysis. J. Bone Joint Surg. Br. 70, 461–464 (1988). 3372571 10.1302/0301-620X.70B3.3372571

[42] M. A. McDowell, C. D. Fryar, C. L. Ogden, Anthropometric reference data for children and adults: United states, 1988–1994. Vital Health Stat. 11 , 1–68 (2009). 19642512

[43] J. D. Rees, A. M. Wilson, R. L. Wolman, Current concepts in the management of tendon disorders. Rheumatology 45, 508–521 (2006). 16490749 10.1093/rheumatology/kel046

[44] A. Jurca, J. Žabkar, S. Džeroski, Analysis of 1.2 million foot scans from North America, Europe and Asia. Sci. Rep. 9, 19155 (2019). 31844106 10.1038/s41598-019-55432-z PMC6914786

[45] C. E. Quenneville, S. D. McLachlin, G. S. Greeley, C. E. Dunning, Injury tolerance criteria for short-duration axial impulse loading of the isolated tibia. J. Trauma Acute Care Surg. 70, E13–E18 (2011). 10.1097/TA.0b013e3181f6bb0e 21217472

[46] C. Loercher, S. Morlock, A. Schenk, Design of a motion-oriented size system for optimizing professional clothing and personal protective equipment. J. Fash. Technol., (2018).

