# Supplementary material for: Slamming dynamics of diving and its implications for diving-related injuries
Source: Sci Adv. 2022 Jul 27;8(30):eabo5888. doi: 10.1126/sciadv.abo5888 (PMC9328685; doi:10.1126/sciadv.abo5888)
Supplement: Supplementary file 1 — Sections SI to SIV Figs. S1 to S5 Table S1 References [file sciadv.abo5888_sm.pdf]

Science Advances
AAAS

# Supplementary Materials for

## Slamming dynamics of diving and its implications for diving-related injuries

Anupam Pandey *et al.*

Corresponding author: Sunghwan Jung, sunnyjsh@cornell.edu

*Sci. Adv.* **8**, eabo5888 (2022)
DOI: 10.1126/sciadv.abo5888

**The PDF file includes:**

Sections SI to SIV
Figs. S1 to S5
Table S1
Legends for movies S1 to S6
References

**Other Supplementary Material for this manuscript includes the following:**

Movies S1 to S6

## I. GEOMETRICAL FEATURES OF THE 3D PRINTED MODELS

Here, we provide the values of the key geometrical features of all six different models tested in this paper. We start with the porpoise head as shown in fig. S1a. Due to the blunt and rounded shape of its snout, we approximate the porpoise head as a paraboloid with a mean base radius $R_s = (d_1 + d_2)/4$ and height $H_s$. Thus, the curvature at the tip of the snout becomes, $\kappa_m = 2H_s/R_s^2 = 1.05$ cm$^{-1}$, where $H_s = 8.4$ cm and $R_s = 4.0$ cm.

The gannet beak (S1b) is approximated as a cone with height $(H_b) = 13.4$ cm and base radius $(R)$ 2.86 cm, leading to a half-solid angle, $\beta$= 0.21 rad.

The lizard foot (S1c) consists of long and slender fingers connected to a small flat region near the heel. We simplify the shape by considering a circular flat plate of equal area. For our 3D printed lizard foot model, the radius $(R)$ of the equivalent circular plate is 1.9 cm.

We print human models in two different sizes; the larger one is 1.5 times larger than the other size. The head-first dive models (S1d) have head radii $(R)$ of 2.06 cm and 3.09 cm.

The hand-first model is approximated as a cone with elliptical cross-section. The half-solid angles along the two principal directions are given by $\beta_1 = \tan^{-1}(w_1/H)$ and $\beta_2 = \tan^{-1}(w_2/H)$, where $w_1$ and $w_2$ are the width and depth of the model at the shoulder (cf. fig. S1e). $\beta_1 = 23\pi/180$ and $\beta_2 = \pi/20$ for both hand-first models with height (H) is equal to 13.4 cm for smaller size. The larger model has a height of 20.1 cm.

We simplify the contact area of the feet-fist model by considering a circular plate of same area. Now the bottom of human foot is not simply flat, rather the bottom surface is concave due to the lateral arch. Thus we approximate the foot area based on the span of the arch which is $0.7L$, where $L$ is the largest between heel to toe. Fig.S1f shows the planar geometry of the foot model where the dimensions (scaled) are consistent with average North American male feet size as given in [42]. Thus the area of the feet becomes by $(w_1 + w_2)\,(0.7L) = 19.32$ cm$^2$, where $w_1 = 2.6$ cm, $w_2 = 2$ cm, $L = 6$ cm, and $L_2 = 5.5$ cm (S1f). Thus, the radius of the equivalent circular plate comes out to be 2.48 cm. For the model of a larger size, this radius is 3.67 cm. The concave nature of the bottom surface of the foot traps an air bubble during impact, and leads to high frequency oscillations in the force data. Natural frequency of a spherical bubble of radius $w_2$ (the smallest dimension) captures these oscillations in the force data.

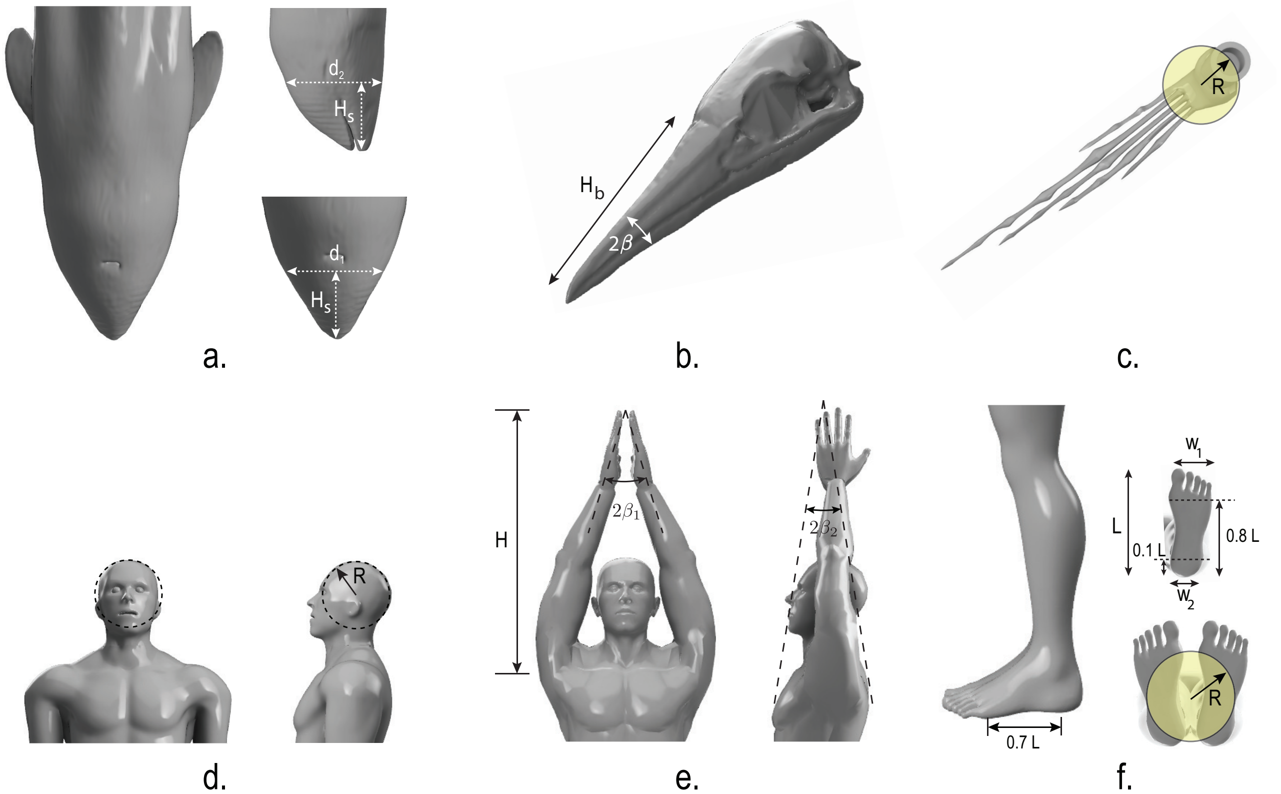


**FIG. S1: Geometry of animal and human models**. a. We characterize the porpoise snout by measuring its height ($H_s$) from the tip to the blowhole, along with the head width at the blowhole in the posterior ($d_1$) and lateral ($d_2$) side. b. The conical gannet head is characterized by the solid angle, $\beta$, and its height, $H_b$. c. The impact area of the lizard foot is primarily localized around the heel which we characterize by the area of an equivalent circular plate of radius $R$. d. Human head is approximated as a sphere of radius $R$. e. We assume the shape of the diving front to be a cone of elliptical cross section in the hand-first case. Thus the shape is characterized by the cone angles in anterior ($\beta_1$) and posterior ($\beta_2$) sides, along with the height $H$. f. The planar dimensions of the foot are used to calculate the radius ($R$) of an equivalent circular plate.

## II. DIMENSIONLESS GROUPS

Table S1 lists the length scales associated to each of the 3D printed models and the three relevant dimensionless groups, Reynolds number (Re), Weber number (We), and Froude number (Fr).

| Model | Length scale ($L$) | Re=$\frac{\rho V L}{\eta}$ | We=$\frac{\rho V^2 L}{\gamma}$ | Fr=$\frac{V}{\sqrt{gL}}$ |
|---|---|---|---|---|
| Porpoise head | Snout height<br>$H$ =8.4 cm | $\geq 1.6 \times 10^5$ | $\geq 4.6 \times 10^3$ | $2.18 - 4.88$ |
| Gannet head | Beak height<br>$H$ =13.4 cm | $\geq 2.65 \times 10^5$ | $\geq 7.3 \times 10^3$ | $1.73 - 3.86$ |
| Lizard foot | Radius<br>$R$ =1.9 cm | $\geq 3.76 \times 10^4$ | $\geq 1.03 \times 10^3$ | $4.58 - 10.26$ |
| Head-first | Head radius | | | |
| Size 1 | $R$ =2.06 cm | $\geq 4.1 \times 10^4$ | $\geq 1.1 \times 10^3$ | $4.41 - 9.85$ |
| Size 2 | $R$ =3.09 cm | $\geq 6.1 \times 10^4$ | $\geq 1.7 \times 10^3$ | $3.60 - 8.05$ |
| Hand-first | Arms height | | | |
| Size 1 | $H$ =13.4 cm | $\geq 2.65 \times 10^5$ | $\geq 7.3 \times 10^3$ | $1.73 - 3.86$ |
| Size 2 | $H$ =20.1 cm | $\geq 3.98 \times 10^5$ | $\geq 1.09 \times 10^4$ | $1.41 - 3.15$ |
| Feet-first | Radius | | | |
| Size 1 | R=2.9 cm | $\geq 5.74 \times 10^4$ | $\geq 1.58 \times 10^3$ | $3.71 - 8.30$ |
| Size 2 | R=4.35 cm | $\geq 8.61 \times 10^4$ | $\geq 2.37 \times 10^3$ | $3.03 - 6.78$ |

**TABLE S1: Values of the relevant dimensionless groups for different models**. Both the Re and We are shown for the lowest velocity of 1.98 m/s which corresponds to a free fall height of 20 cm.

## III. DIMENSIONAL FORCE DATA

In this section, we show the dimensional force versus time data for the animal and human models.

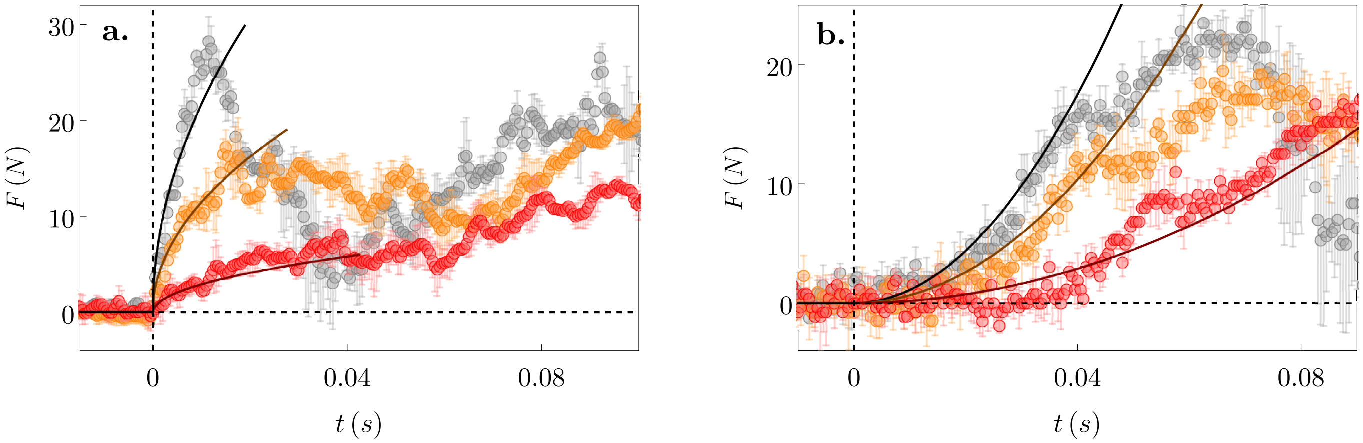


**FIG. S2: Slamming force versus time curve for porpoise snout (a) and gannet beak (b) at different impact velocities**. a. The data points represent porpoise snout impacting water at 4.41 m/s (gray), 3.42 m/s (orange), and 1.98 m/s (red). The solid lines are predictions of Eq.(2) of the main manuscript. b. Force data for gannet beaks at impact speeds of 4.41 m/s (gray), 3.95 m/s (orange), and 2.80 m/s (red). Eq.(3) of the main manuscript is plotted as the solid lines corresponding to each velocity. The data points represent mean values over five trials, and the error bars represent standard deviation.

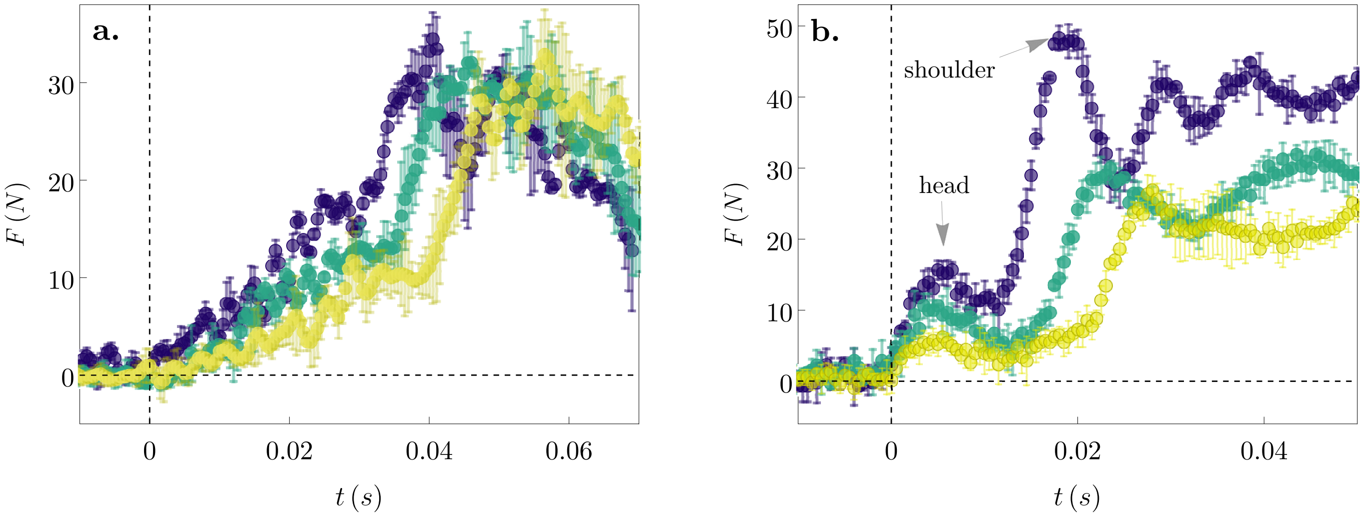


**FIG. S3: Slamming force versus time curve for hand-first (a) and head-first (b) dives at different impact velocities**. a. The data points represent hand-first dives at 4.41 m/s (blue), 3.95 m/s (green), and 3.43 m/s (yellow). b. Force data for head-first dives at 4.41 m/s (blue), 3.43 m/s (green), and 2.80 m/s (yellow). The first peak corresponds to the maximum slamming force on head while the second peak is due to impact of the shoulder on to water surface.

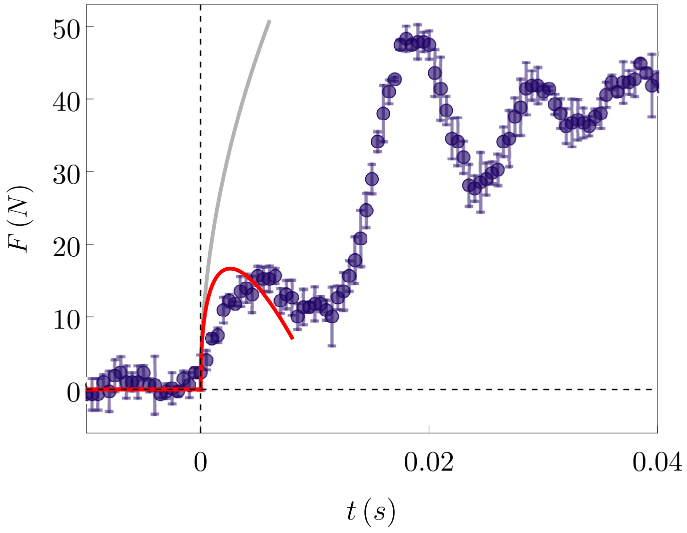


**FIG. S4: Comparison between experiment and theory for the head-first dive**. The data points represent experiment for an impact speed of 4.43 m/s.The gray line is the prediction of Eq.(2) of the main manuscript, $F = 4\sqrt{2}\rho\kappa_m^{-3/2}V^{5/2}t^{1/2}$ which overestimates the force. The red line represents Eq.(4) of the manuscript, $F = 4\sqrt{2}\rho\kappa_m^{-3/2}V^{5/2}t^{1/2} - 1.19\pi\rho\kappa_m^{-1}V^3t$ . It captures the non-monotonic nature of the force as well as the peak force on the head.

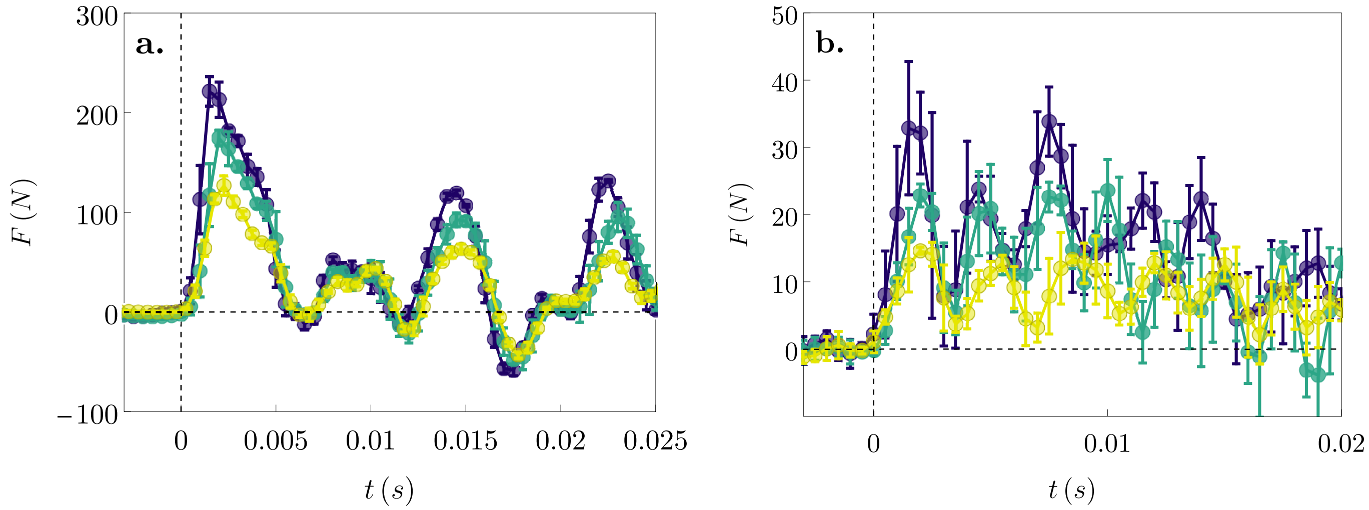


**FIG. S5: Slamming force versus time curve for human (a) and lizard feet (b) at different impact velocities**. a. The data points represent feet-first dives at 3.95 m/s (blue), 3.43 m/s (green), and 2.80 m/s (yellow). b. Force data for lizard foot impact at 3.43 m/s (blue), 2.80 m/s (green), and 1.98 m/s (yellow).

## IV. TIME-AVERAGED IMPULSE

In this section we present the explicit evaluation of Eq.(6) of the main manuscript that leads to the shape factor ($\alpha$) for the head-first, hand-first, and feet-first dives , and discuss the relevant sizes ($L$) of average human body that connects our experimental results to critical force of injury.

Since the force data in head-first dives are well captured by Eq. (3) of the manuscript, the time-averaged impulse results in

$$\begin{aligned}\langle F_s\rangle &= \frac{8\sqrt{2}}{3}\rho R^{3/2}V^{5/2}t_s^{1/2} - \frac{1.19\pi}{2}\rho R V^3 t_s \\ &= \left(\frac{8\sqrt{2}}{3} - \frac{1.19\pi}{2}\right)\rho R^2 V^2 \qquad \text{(since } t_s = R/V\text{)} \\ &= \left(\frac{16\sqrt{2}}{3} - 1.19\pi\right)\rho g R^2 h \qquad \text{(plugging } V^2 = 2gh\text{)}.\end{aligned}$$

Here we denote the numerical pre-factor to be $\alpha$ as given in the Table 1 of the manuscript. Recaling the diving height $h$ by head radius $R(= L)$, and $\langle F_s\rangle$ by $\alpha\rho g L^3$, we get $\langle \overline{F_s}\rangle = h$. We incorporate the critical spine and skull injury forces in Fig. 4b by rescaling the force values by $\alpha\rho g L^3$, where $L$ is taken to be the average head radius of 9.02 cm [35]. Thus for the head-first dive,

$$R = L = 9.02 \text{ cm} \quad \text{and} \quad \alpha = \left(\frac{16\sqrt{2}}{3} - 1.19\pi\right).$$

We simplify the morphology of the hand-fist dive as a cone with ellipsoidal cross-section of mean radius $r = z(\tan\beta_1 \tan\beta_2)^{1/2}$, where angles $\beta_1$ and $\beta_2$ are shown fig. S2 e. Thus Eq.(6) of the manuscript becomes

$$\begin{aligned}\langle F_s\rangle &= \frac{\pi}{3}\rho V^4 (\tan\beta_1\tan\beta_2)^{3/2} t_s^2 \\ &= \frac{2\pi}{3}\rho g H^2 (\tan\beta_1\tan\beta_2)^{3/2} h \qquad (\text{using}\, t_s = R/V\,, V^2 = 2gh) \\ &= \frac{2\pi}{3}(\tan\beta_1\tan\beta_2)^{1/2}\rho g w_m^2 h.\end{aligned}$$

Here $w_m = H(\tan\beta_1\tan\beta_2)^{1/2}$ is the mean shoulder half-width. In this case we define the shape factor $\alpha = 2\pi/3\,(\tan\beta_1\tan\beta_2)^{1/2}$. To estimate these parameters for human body, we assume that $\beta_1$ and $\beta_2$ remain constant, as 3D printed models are scaled down version of typical human body. Using an average arm length of 76 cm [48, 39] and $\beta_1 = 23\pi/180$, $\beta_2 = \pi/20$ from our model, we get

$$w_m = L = 18.14 \text{ cm} \quad \text{and} \quad \alpha = 0.54.$$

The evolution of feet-first force data is not captured by an analytical force-time relation. Thus we simply chose a force scale of $\alpha\rho g R^3$, where $R$ is the radius of an equivalent circular plate having same area as the feet. Analogous to section 2, using average feet dimensions [42], we find the equivalent radius which along the shape factor ($\alpha$) that is chosen from literature [29] are given by

$$L = 7.04 \text{ cm} \quad \text{and} \quad \alpha = 11.$$

## V. SUPPLEMENTARY MOVIES

**Movie S1.avi: Diving porpoise head from a height of 50 cm**.

**Movie S2.avi: Diving gannet head from a height of 50 cm**.

**Movie S3.avi: Impact of lizard foot from a height of 50 cm**.

**Movie S4.avi: Human head-first dive from a height of 50 cm**.

**Movie S5.avi: Human hand-first dive from a height of 50 cm**.

**Movie S6.avi: Human feet-first dive from a height of 50 cm**.

## REFERENCES AND NOTES


1. C. Clanet, F. Hersen, L. Bocquet, Secrets of successful stone-skipping. *Nature* **427**, 29 (2004).

2. T. Truscott, J. Belden, R. Hurd, Water-skipping stones and spheres. *Phys. Today* **67**, 70–71 (2014).

3. J. G. Brown, L. D. Abraham, J. J. Bertin, Descriptive analysis of the rip entry in competitive diving. *Res. Q. Exerc. Sport* **55**, 93–102 (1984).

4. H. Driscoll, S. Gaviria, S. Goodwill, Analysing splash in competitive diving. *Procedia Eng.* **72**, 26–31 (2014).

5. S. Garthe, S. Benvenuti, W. A. Montevecchi, Pursuit plunging by northern gannets (*Sula bassana*)" feeding on capelin (*Mallotus villosus*)". *Proc. R. Soc. B Biol. Sci.* **2670**, 1717–1722 (2000).

6. B. Chang, M. Croson, L. Straker, S. Gart, C. Dove, J. Gerwin, S. Jung, How seabirds plunge-dive without injuries. *Proc. Natl. Acad. Sci.* **113**, 12006–12011 (2016).

7. A. May, J. C. Woodhull, Drag coefficients of steel spheres entering water vertically. *J. Appl. Phys.* **19**, 1109–1121 (1948).

8. A. May, J. C. Woodhull, The virtual mass of a sphere entering water vertically. *J. Appl. Phys.* **21**, 1285–1289 (1950).

9. T. Von Karman, *The Impact on Seaplane Floats During Landing* (National Advisory Committee on Aeronautics, 1929).

10. J. W. Glasheen, T. A. McMahon, A hydrodynamic model of locomotion in the basilisk lizard. *Nature* **380**, 340–342 (1996).

11. T. Guillet, M. Mouchet, J. Belayachi, S. Fay, D. Colturi, P. Lundstam, P. Hosoi, C. Clanet, C. Cohen, The hydrodynamics of high diving. *Proceedings* **49**, 73 (2020).

12. R. Labbé, J.-P. Boucher, C. Clanet, M. Benzaquen, Physics of rowing oars. *New J. Phys.* **21**, 093050 (2019).

13. T. T. Truscott, B. P. Epps, J. Belden, Water entry of projectiles. *Annu. Rev. Fluid Mech.* **46**, 355–378 (2014).

14. K. Bhar, B. Chang, E. Virot, L. Straker, H. Kang, R. Paris, C. Clanet, S. Jung, How localized force spreads on elastic contour feathers. *J. R. Soc. Interf.* **16**, 20190267 (2019).

15. N. S. Jones, Competitive diving principles and injuries. *Curr. Sports Med. Rep.* **16**, 351–356 (2017).

16. S. M. Harrison, R. C. Z. Cohen, P. W. Cleary, S. Barris, G. Rose, Forces on the body during elite competitive platform diving, in *Ninth International Conference on CFD in the Minerals and Process Industries* (CSIRO, 2012), Melbourne, Australia, 10 to 12 December 2012.

17. D. Wharton, Broken wrists, twisted necks and concussions: The brutal nature of olympic diving, *Los Angeles Times*, 29 July 2021.

18. S. C. Haase, Management of upper extremity injury in divers. *Hand Clin.* **33**, 73–80 (2017).

19. D. T. le Viet, L. A. Lantieri, S. M. Loy, Wrist and hand injuries in platform diving. *J. Hand Surg. Am.* **18**, 876–880 (1993).

20. S. Abrate, Hull slamming. *Appl. Mech. Rev.* **64**, 060803 (2011).

21. S. Jung, Swimming, flying, and diving behaviors from a unified 2d potential model. *Sci. Rep.* **11**, 15984 (2021).

22. H. Wagner, Phenomena associated with impacts and sliding on liquid surfaces. *J. Appl. Math. Mech.* **12**, 193–215 (1932).

23. T. Miloh, On the initial-stage slamming of a rigid sphere in a vertical water entry. *Appl. Ocean Res.* **13**, 43–48 (1991).

24. A. A. Korobkin, V. V. Pukhnachov, Initial stage of water impact. *Annu. Rev. Fluid Mech.* **20**, 159–185 (1988).

25. M. Moghisi, P. T. Squire, An experimental investigation of the initial force of impact on a sphere striking a liquid surface. *J. Fluid Mech.* **108**, 133–146 (1981).

26. F. J. Huera-Huarte, D. Jeon, M. Gharib, Experimental investigation of water slamming loads on panels. *Ocean Eng.* **38**, 1347–1355 (2011).

27. V. Mathai, R. N. Govardhan, V. H. Arakeri, On the impact of a concave nosed axisymmetric body on a free surface. *Appl. Phys. Lett.* **1060**, 064101 (2015).

28. M. Minnaert. On musical air-bubbles and the sounds of running water. *Lond. Edinb. Dublin Philos. Mag. J. Sci.* **16**, 235–248 (1933).

29. A. K. D. Nguyen, A. A. Simard-Meilleur, C. Berthiaume, R. Godbout, L. Mottron, Head circumference in Canadian male adults: Development of a normalized chart. *Int. J. Morphol.* **30**, 1474–1480 (2012).

30. Y. Ropert-Coudert, D. Grémillet, P. Ryan, A. Kato, Y. Naito, Y. L. Maho, Between air and water: The plunge dive of the Cape gannet *Morus capensis*. *Ibis* **146**, 281–290 (2004).

31. S. I. Sharker, S. Holekamp, M. M. Mansoor, F. E. Fish, T. T. Truscott, Water entry impact dynamics of diving birds. *Bioinspir. Biomim.* **140**, 056013 (2019).

32. D. N. Lee, P. E. Reddish, Plummeting gannets: A paradigm of ecological optics. *Nature* **293**, 293–294 (1981).

33. E. A. Buchholtz, S. A. Schur, Vertebral osteology in delphinidae (cetacea). *Zool. J. Linn. Soc.* **140**, 383–401 (2004).

34. F. E. Fish, C. A. Hui, Dolphin swimming—A review. *Mamm. Rev.* **21**, 181–195 (1991).

35. A. E. Minetti, Y. P. Ivanenko, G. Cappellini, N. Dominici, F. Lacquaniti, Humans running in place on water at simulated reduced gravity. *PLOS ONE* **7**, e37300 (2012).

36. D. S. Korres, I. S. Benetos, G. S. Themistocleous, A. F. Mavrogenis, L. Nikolakakos, P. T. Liantis, Diving injuries of the cervical spine in amateur divers. *Spine J.* **6**, 44–49 (2006).

37. T. Whyte, A. D. Melnyk, C. Van Toen, S. Yamamoto, J. Street, T. R. Oxland, P. A. Cripton, A neck compression injury criterion incorporating lateral eccentricity. *Sci. Rep.* **10**, 7114 (2020).

38. F. Li, N.-s. Liu, H.-g. Li, B. Zhang, S.-w. Tian, M.-g. Tan, B. Sandoz, A review of neck injury and protection in vehicle accidents. *Transp. Saf. Environ.* **1**, 89–105 (2019).

39. S. Advani, W. Powell, J. Huston, S. Ojala, Human head impact response–experimental data and analytical simulations, in *Proceedings of the International Conference on the Biomechanics of Impact* (IRCOBI, 1975), pp. 153–162.

40. D. C. Schneider, A. M. Nauhm, Impact studies of facial bones and skull, in *Proceedings: Stapp Car Crash Conference* (Society of Automotive Engineers SAE, 1972), vol. 16.

41. D. Stanley, E. A. Trowbridge, S. H. Norris, The mechanism of clavicular fracture. A clinical and biomechanical analysis. *J. Bone Joint Surg. Br.* **70**, 461–464 (1988).

42. M. A. McDowell, C. D. Fryar, C. L. Ogden, Anthropometric reference data for children and adults: United states, 1988–1994. *Vital Health Stat. 11*, 1–68 (2009).

43. J. D. Rees, A. M. Wilson, R. L. Wolman, Current concepts in the management of tendon disorders. *Rheumatology* **45**, 508–521 (2006).

44. A. Jurca, J. Žabkar, S. Džeroski, Analysis of 1.2 million foot scans from North America, Europe and Asia. *Sci. Rep.* **9**, 19155 (2019).

45. C. E. Quenneville, S. D. McLachlin, G. S. Greeley, C. E. Dunning, Injury tolerance criteria for short-duration axial impulse loading of the isolated tibia. *J. Trauma Acute Care Surg.* **70**, E13–E18 (2011).

46. C. Loercher, S. Morlock, A. Schenk, Design of a motion-oriented size system for optimizing professional clothing and personal protective equipment. *J. Fash. Technol.*, (2018).
